# Development of a high yield expression and purification system for Domain I of Beta-2-glycoprotein I for the treatment of APS

**DOI:** 10.1186/s12896-015-0222-0

**Published:** 2015-11-14

**Authors:** Thomas McDonnell, Charis Pericleous, Emmanuelle Laurine, Rita Tommasi, Acely Garza-Garcia, Ian Giles, Yiannis Ioannou, Anisur Rahman

**Affiliations:** Centre for Rheumatology, Division of Medicine, University College London, Rayne Institute, 5 University Street, London, WC1E 6JF UK; PolyTherics, Babraham Research Campus, Babraham, CB22 3AT Cambridge, UK; Structural Biology, Medical Research Council National Institute for Medical Research, London, UK; Arthritis Research UK Centre for Adolescent Rheumatology, University College London, London, UK

**Keywords:** Antiphospholipid syndrome, Protein production, Inclusion bodies, E. Coli, Beta-2-Glycoprotein I, Domain I, Automated

## Abstract

**Background:**

In this paper we describe a novel method to achieve high yield bacterial expression of a small protein domain with considerable therapeutic potential; Domain I of Beta-2-glycoprotein I (β2GPI). β2GPI is intrinsic to the pathological progression of the Antiphospholipid Syndrome (APS). Patients develop autoantibodies targeting an epitope located on the N-terminal Domain I of β2GPI rendering this domain of interest as a possible therapeutic.

**Results:**

This new method of production of Domain I of β2GPI has increased the production yield by ~20 fold compared to previous methods in E.coli. This largely scalable, partially automated method produces 50–75 mg of pure, folded, active Domain I of β2GPI per litre of expression media.

**Conclusion:**

The application of this method may enable production of Domain I on sufficient scale to allow its use as a therapeutic.

## Background

Protein-based biologic agents are increasingly used in the treatment of a range of autoimmune diseases. They may exert their effects by blocking receptor-ligand or antibody-antigen interactions or by delivering an exogenous enzyme such as uricase [1–5]. It is necessary however, to modify small proteins in order to create optimal therapeutic agents in particular to increase their in-vivo half-life and reduce their immunogenicity. PEGylation is one of the main methods used to achieve this and PEGylated proteins currently used in clinical practice include Puricase® (PEG-uricase) for gout [2, 6], PegIntron®/PEGASYS® (PEG-Interferon alpha) for hepatitis C and myeloid leukemia [7, 8] and Cimzia® (certolizumab pegol) for rheumatoid arthritis [9–11]. The production of pure active PEGylated protein requires significant amounts of native human protein as raw material for PEGylation and extraction of these amounts from blood samples of human volunteers is not feasible. It is therefore critical to obtain large scale production of recombinant soluble, folded protein to be used as starting material for PEGylation. Mammalian, insect, yeast and bacterial expression systems could all be used to approach this goal.

The advantages of heterologous protein expression in *Escherichia coli* are many fold: (I) the technique is well studied and established, (II) yields per volume of culture are potentially very high, (III) the materials and carbon sources needed for cell growth and protein expression are inexpensive and, (IV) a variety of E coli-expressed proteins suitable for human use have already been commercialised [12–14]. There are, however, also significant drawbacks to bacterial expression. One of the most significant is that recombinant proteins are often misfolded and/or aggregated and found in insoluble particles called inclusion bodies. Refolding aggregated protein is a complex process resulting in low yields [15, 16]. Another limitation of expression in the bacterial cytoplasm is the impossibility of obtaining proteins with post-translational modifications such as disulphide bond formation or glycosylation. In some cases, this limitation can be circumvented by directing the expression product to the bacterial periplasm where some post-translational modifications naturally occur. Also, advances have been made in the design of genetically engineered bacterial strains with enhanced post-translational modification capabilities [17–19]. The final consideration when proteins are produced in *E. coli* is contamination of the purified protein with Gram-negative bacterial cell wall components. The presence of these endotoxins precludes the use of the preparation in vivo or for cell culture studies.

Mammalian cell expression has some advantages over bacterial expression, mainly lack of endotoxin contamination and production of proteins with native or near-native post-translational modifications. Yields tend to be lower than in bacterial expression, but methods have been developed to increase them [20], The main disadvantages of expression in mammalian cells are that it requires very specialised equipment [20, 21] is expensive -at least in a bench top scale-, and expression levels rely heavily on a random integration process [21]. Similarly some groups use insect cells and yeasts such as *Pichia pastoris.* However, these systems are less well-developed, can be delicate to manipulate, lower yields are frequent and yeast tends to generate escape mutants in culture [22]. It is for these reasons, cost, efficiency, flexibility and convenience, that most often laboratory protein expression prefers to utilize bacterial hosts.

In this paper we describe the development of a novel method for medium scale bacterial expression of a small protein domain with considerable therapeutic potential, the N-terminal domain of beta-2-glycoprotein I (β2GPI), commonly designated domain I (DI). DI is a critical antigen in the anti-phospholipid syndrome (APS).

APS is an autoimmune disease characterised by vascular thrombosis and/or recurrent miscarriages in patients and is a significant cause of mortality and morbidity. It is also the leading cause of strokes in patients under 50 years of age [23–25]. The disease is characterised by the presence of a heterogeneous population of auto-antibodies [26] that bind a range of antigens, in particular beta-2-glycoprotein I (β2GPI) [25, 27–29]. Anti-β2GPI antibodies have been closely associated with thrombosis implying a significant role of these antibodies in the pathogenesis of the disease [30, 31]. Current treatment for APS patients is long-term anticoagulation with warfarin or heparin, which are a non-specific vitamin K dependent coagulation blocking agent and an activator of anti-thrombin III respectively. These treatments lack efficacy in some cases and carry a significant risk of side-effects such as haemorrhage [32]. There is thus a pressing need to develop new targeted therapies such as drugs that would inhibit binding of anti-β2GPI antibodies to β2GPI. The critical pathogenic epitope of β2GPI [25, 29, 33, 34] has been defined as a conformational epitope covering residues 8 and 9 and 39–43 on DI. Recombinant DI inhibits the binding of antibodies derived from APS patients to β2GPI in ELISA binding assay [25]. In vivo studies showed that recombinant DI inhibits the development of thrombosis in mice exposed to IgG from patients with APS [23]. DI is non-glycosylated and is thus ideally suited for bacterial expression. We have also used recombinant DI as the substrate in an ELISA to detect serum anti-DI antibodies. In studies of large numbers of sera from patients with APS, autoimmune disease controls and healthy controls, anti-DI positivity enhances the ability to differentiate patients with APS from other groups (reviewed in Bertolaccini et al. [35]).

In previously published work we developed two methods for the expression of DI in *E. coli*. The yield of soluble hexa-histidine tagged DI was around 0.75 mg/L of bacterial culture [36] when the expression product was targeted to the bacterial periplasm and approximately 4 mg/L when recovered from inclusion bodies [37]. Now we present a novel method suitable for medium-scale production of highly pure human DI in *E.coli*, and verify protein activity by ELISA assays showing that the expression product inhibits binding of IgG from patients with APS to β2GPI.

## Methods

All chemicals were purchased from Sigma, unless otherwise stated.

### DI expression vector

**Fig. 1:**
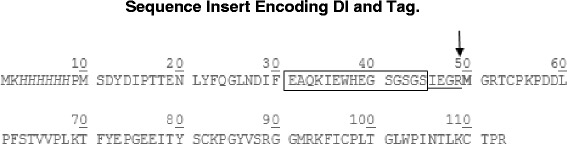
DI fusion protein sequence showing the N-terminal hexa-histidine tag (italics), the BirA recognition site for potential biotinylation (boxed) and the restriction protease FXa cleavage site (underlined). The cleavage site is indicated with an arrow and the first amino acid of DI is bolded. The fusion protein is 12.9 kDa while native DI which contains 64 amino acids is 7.2 kDa

The synthetic DI coding sequence as previously published [37] was cloned into an in-house modified vector that incorporates an N-terminal fusion tag consisting of an hexa-histidine motif for purification and expression, a BirA biotinylation site and a Factor Xa recognition sequence for proteolytic cleavage (Fig. 1). These modifications were introduced into pETHis_1a (a kind gift from Gunter Stier, then at EMBL Heidelberg), which is itself a modified pET24d vector (Merck Millipore). The biotinylation site was introduced during the development of the vector to allow the option of binding to streptavidin-coated plates in ELISA, but we are not using biotinylated DI for any purpose at present.

### Small scale protein expression test in Luria Broth (LB) and Terrific Broth (TB)

Transformation of BL21*DE3® (Life Technologies) cells was carried out according to the manufacturer’s instructions. Cells were plated into LB/agar with kanamycin 50 μg/ml. Single colonies were picked and subcultured into 3 ml of LB media containing kanamycin (50 μg/ml) and incubated overnight at 250 rpm and 37 °C. 150 μl of preculture were used to inoculate 3 ml of LB or TB and cells were incubated at 250 rpm and 37 °C; growth was monitored by OD at 600 nm. For cultures in LB, expression was induced with 1 mM IPTG at an OD_600nm_ of 0.6. In order to compare different expression temperatures, the induced culture was incubated overnight with shaking at 250 rpm at either 20 °C or 37 °C. For cultures in TB, expression was induced with 1 mM IPTG at an OD_600nm_ of between 5 and 7. Post induction, expression was continued overnight with shaking at 250 rpm at 20 °C. Cells were harvested by centrifugation for 20 min at 20,000 x g and 4 °C. Pellets were lysed in 500 μl of 1 % Triton x-100 and 1x PBS, by three cycles of sonication (output 100, 3 min). Samples were analysed by SDS PAGE.

### Large scale protein expression in TB

A glycerol stock was used to inoculate 200 ml of LB. Cultures were incubated overnight with shaking at 225 rpm and 37 °C, centrifuged at 3500 × g for 30 min at room temperature and resuspended in 20 ml of fresh LB. Two litres of TB were seeded in four 2 L-flasks with 5 ml of pre-culture in each. Expression was then carried out as for the small scale protocol.

### Harvesting

Harvesting was carried out using a 500 kDa Hollow Fiber Ultrafiltration Cartridge (GE Healthcare). The cell suspension was run into the cartridge at 100 rpm until a pellet was formed and the bacterial pellet was further rinsed by addition of 2 L of PBS. The pellet was transferred into 50 ml centrifuge tubes, spun at 3,500 × g for 30 min to remove any remaining PBS and finally snap frozen using dry ice.

### Cell lysis and inclusion body solubilisation

Lysis Buffer (A; 50 mM sodium phosphate, 0.3 M Sodium Chloride, 10 mM Imidazole) was added to the frozen pellet with the addition of DNase (0.02 mg/ml) and protease inhibitors (1:500). Bacterial pellets were suspended by vortexing and pipette mixing. The lysate was then sonicated (50 % maximum intensity, 50 % cycles) for 4 min, allowed to cool for 2 min. This was repeated once. The inclusion bodies were collected by centrifugation of the lysate at 3,500 × g for 30 min. Lighter inclusion bodies were harvested by spinning the supernatant once again at 20,000 × g for 30 min.

### Inclusion body preparation

The inclusion bodies were resuspended in Solubilisation Buffer (B; 6 M guanidine hydrochloride, 0.1 M NaH_2_PO_4_, 10 mM Tris, pH 8.0) initially by grinding in a pestle and mortar homogeniser. The suspension was sonicated twice for 4 min (50 % maximum intensity, 50 % cycles), in order to promote protein solubilisation and reduce viscosity. The suspension was finally centrifuged at 20,000 × g for 20 min to remove insoluble debris prior to purification.

### Purification of denatured protein by immobilised metal affinity chromatography (IMAC)

The solubilised protein was loaded at 2–5 ml/min onto a 15-ml HisTrap FF (GE Healthcare) and washed with 1.5 column volumes (CV) of Denaturing IMAC Equilibration Buffer (C; 6 M guanidine hydrochloride, 0.1 M NaH_2_PO_4_, 10 mM Tris, pH 6.3). The protein was eluted at 1 ml/min with 2.7 CV of Denaturing IMAC Elution Buffer (D; 6 M guanidine hydrochloride, 0.1 M NaH_2_PO_4_, 10 mM Tris, pH 4.5). The fractions were collected and analysed by SDS-PAGE. The entire purification process was conducted on an AKTA platform (GE Healthcare).

### In vitro oxidative protein folding

The protein was pooled and concentrated to 25–30 mg/ml by centrifugal concentrators. Any potential disulphide bonds were reduced by incubating overnight with 50 mM TCEP at 4 °C. TCEP was subsequently removed by buffer exchange using a PD-10 desalting column (GE Healthcare). The protein was recovered at a concentration of >10 mg/ml and was loaded into a 5 ml syringe. This syringe was fitted into a pump with the tip placed inside 95 mL of the Folding Buffer (E; 0.6 M arginine hydrochloride, 0.1 M Tris, pH 8.5, 1 mL cysteine (0.3 M) + 1 mL cystine (0.03 M). The pump was set to inject constantly at 300 μl/h and left overnight at 4 °C. The resulting solution was centrifuged at 3,500 × g for 30 min at 4 °C and syringe filtered (25 μm, Sartorius). The protein solution was further concentrated to approximately 3–5 mg/ml by Vivaspin® centrifugal ultrafiltration (5 kDa MWCO, Satorius) and dialysed for 2 h (3.5 KDa MWCO;, snake skin, thermo scientific) against 5 L of Native IMAC Equilibration Buffer (F; 20 mM Tris, 0.1 M NaCl, pH 8.0) at 4 °C. Dialysis buffer was changed after 2 h and the protein was further dialysed overnight at 4 °C. The resultant protein was collected and used for purification by IMAC.

### Isolation of folded and monomeric protein by IMAC in native conditions

Dialysed protein was purified using a 15-ml HisTrap FF (GE Healthcare) at a flow rate of 2 ml/min. The flow-through was collected and the column washed with 3–4 CV of Buffer F. The protein was eluted with a gradient 0–100 % Native IMAC Elution Buffer (G; 20 mM Tris, 0.1 M NaCl, 1 M imidazole, pH 8.0) over 8 CV (2 ml/min). Five millilitre fractions were collected and analysed by SDS PAGE. The fractions containing recombinant his-tagged DI were pooled and stored at 4 °C.

### Fusion tag cleavage and purification of untagged protein

Purified recombinant tagged DI was dialysed against PBS (~30 ml in 2 L of PBS for 2 h at 4 °C then the buffer was exchanged for 2 L of fresh PBS) and freeze-dried over 48 h in 1 or 2 mg aliquots. Lyophilised recombinant tagged DI was re-suspended in Cleavage Buffer (H; 50 mM Tris, 0.1 M NaCl, 1 mM CaCl_2_, pH 6.5) and human FXa (Haematologic Technologies) was added at a ratio of 1:200 to protein (i.e. 1 μg enzyme per 200 μg protein), protein concentration was kept below 1 mg/ml to avoid aggregation. Calcium chloride was added to a final concentration of 2 mM and the mixture was incubated without shaking for ~16 h at 22 °C. Samples were analysed by SDS-PAGE to assess the completion of cleavage.

The cleaved protein was loaded onto a 5 ml HiTrap SP HP column (GE Healthcare) pre-equilibrated with 3–5 CV of IEX Equilibration Buffer (I; 20 mM HEPES, pH 6.8). After sample loading the column was further washed with 3 CV of Buffer I and the sample was eluted with a 0–100 % linear gradient of IEX Elution Buffer (J: 20 mM HEPES, 1 M sodium chloride, pH 6.8). Fractions were collected, analysed by SDS-PAGE and relevant fractions were pooled, dialysed against PBS at 4 °C (2 L for 2 h, 1 change, and 2 L for 16 h), aliquoted and freeze dried prior to storage at−80°.

### Reverse phase HPLC

Both VariTide RPC (Agilent) and Poroshell C8 (Agilent) columns were used for RP-HLPC. Ten-μg samples dissolved in solvent A were injected and run across either a 4 or a 10 min gradient of 0–100 % solvent B. Solvent A was 2 % acetonitrile and 0.065 % TFA in ddH_2_O and solvent B was 100 % acetonitrile and 0.05 % TFA. Spectra were analysed using Chromeleon® software (Dionex).

### Endotoxin removal and quantification

EndoTrap® high capacity columns (Hyglos) were used for endotoxin removal following the manufacturer’s instructions with the following adaptations: protein was dialysed against PBS overnight before being dialysed into endotoxin-free PBS via centrifugal ultrafiltration. The buffer was supplemented with 1 mM calcium chloride.

The EndoLISA® fluorescence assay (Hyglos) was used for endotoxin quantification following the instructions of the manufacturer. The amount of endotoxin was quantified in protein samples at concentrations between 0.1 mg/ml and 2 mg/ml.

### Competitive inhibition ELISA

Competitive inhibition ELISA was carried out to test the ability of recombinant DI to inhibit binding of purified IgG from patients with APS to immobilised β2GPI. The assay was adapted from the standard β2GPI ELISA published by Ioannou et al. [36] with the following amendments: the inhibitors were pre-incubated at concentrations ranging from 0 mg/ml to 200 μg/ml rather than 0 to 30 μM. Briefly, the test was carried out to test the ability of recombinant DI to inhibit binding of purified IgG from patients with APS to β2GPI coated on a plate. Recombinant DI was pre-incubated with patient serum prior to addition on the ELISA plate coated with β2GPI. Bound patient IgG was detected with an anti-human IgG HRP secondary antibody. Each sample was tested in duplicate.

Ethical approval for use of samples from patients in this research was granted by the London Hampstead Research Ethics Committee Reference Number 12/LO/0373. Patients gave informed consent for use of their samples.

## Results and discussion

### DI fusion protein expression

DI was expressed as a fusion protein containing an additional N-terminal tag including a hexahistidine tag for purification, a biotinylation site and a FXa cleavage motif (Fig. 1). A FXa recognition site was chosen over other more common proteases because no extra amino acids remain in the recombinant protein after cleavage.

**Fig. 2:**
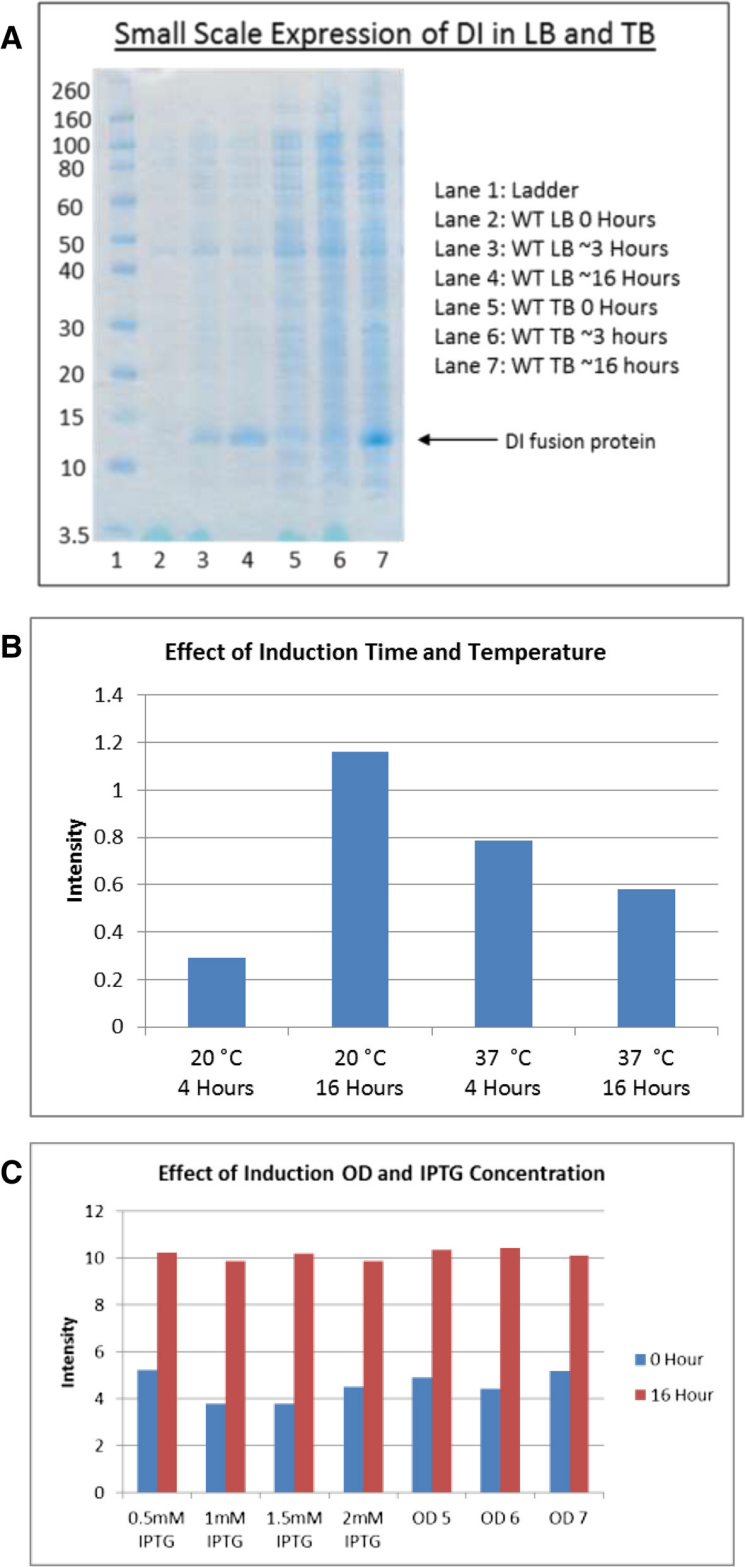
**a** SDS-PAGE gel comparing crude lysates of small scale expression of tagged Domain I fusion protein in LB and TB media before and after induction. Recombinant tagged DI fusion is indicated by an arrow and migrates at 12 kDa. Samples were lysed using triton/PBS and diluted in PBS (5x) and 20 μl was loaded onto an SDS PAGE Gel. The gel was then run, washed briefly in ddH2O and stained for 1 h with InstantBlue™ stain (Expedeon, UK). **b** & **c** Densitometric analysis was carried out on LB and TB samples on a small scale loaded in an identical way, gels were scanned, images converted to TIFF files and analysed. Values represent a calculation of intensity divided by area, OD denotes optical density of cultures at 600 nm at induction

In order to optimise the conditions for the protein expression preliminary small scale expression tests were performed in 24-well plate format. The following conditions were tested: Type of medium (LB or TB), IPTG concentration (0.5 mM, 1 mM or 2 mM), length of incubation after induction (8 or 16 h) and temperature of incubation after induction (20 °C or 37 °C). At the end of the incubation, bacteria were pelleted, lysed and the crude extract was analysed by SDS-PAGE in order to assess the level of protein expression (Fig. 2). Growth in TB with induction at an OD_600_ between 5 and 7 with 1 mM IPTG for 16 h 20 °C was selected as the best condition for the expression of DI fusion protein (Fig. 2); IPTG at 0.5 mM could also be used in the future for larger scale production, as within the tested range, the concentration of IPTG had no impact on the level of expression. No changes in expression level were observed when the production was scaled up to 2 l. Using precultures with an OD_600 nm_ between 0.1 and 0.2, the expression would reach induction density (OD_600nm_ 5–7) within 5 h and reach an overnight OD_600_ of approximately 10–13. Bacteria were harvested by either centrifugation or hollow fiber ultrafiltration. For convenience and suitability for scaling up, ultrafiltration was selected for large scale productions. Typical expression yields were in the order of 20 g of wet cell pellet per litre of culture.

### DI in vitro folding and purification

#### Cell lysis and inclusion body solubilisation

Bacterial pellets were lysed as described in the Methods. Briefly, the bacterial pellets were resuspended in Lysis buffer and subjected to two cycles of sonication. Optimal lysis of the bacterial pellet was achieved using 80 to 100 mL of Buffer A per 40 g of wet cell pellet. Addition of DNase and protease inhibitors in the lysis buffer led to a marked decrease in viscosity of the lysate and an increase in solubility of inclusion bodies at the solubilisation step. The inclusion bodies were collected by centrifugation, re-suspended in Buffer B and homogenised by a combination of grinding with a pestle and mortar, sonicating and pipette mixing; sonication was essential for solubilisation of the inclusion bodies containing DI fusion protein while the incubation temperature had no impact on the solubilisation efficiency. Although using more Buffer B had no impact on the solubilisation yield, less concentrated preparations were less viscous, facilitating subsequent procedures.

#### Purification of denatured DI by IMAC

**Fig. 3:**
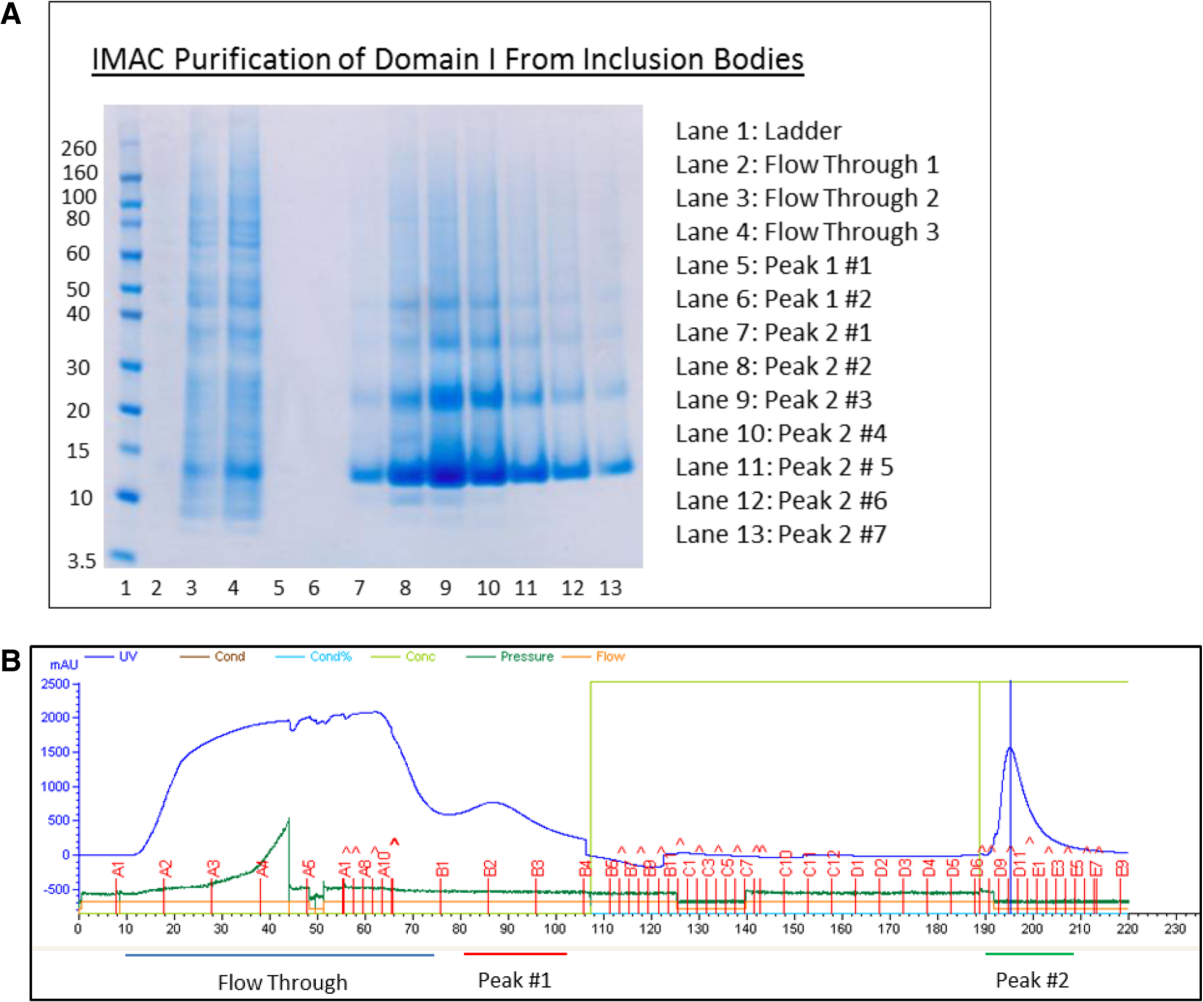
**a** Analysis of denaturing IMAC elution fractions by SDS–PAGE. Samples were diluted 1:8 in water and 15 μl of each dilution were loaded per well. **b** An accompanying chromatogram with two peaks highlighted at the bottom. Peak #1 shows no protein on the gel (lanes 5 and 6) as the amount of this protein was insignificant after dilution to remove guanidine

Solubilised protein from the inclusion bodies was purified by IMAC. DI fusion protein was eluted with a gradient of pH and elution fractions were analysed by SDS-PAGE (Fig. 3). The flow-through still contained a limited amount of DI fusion protein (Lanes 2–4). Elution fractions showed a high concentration of DI fusion protein at ~12 kDa as well as dimers (~24 kDa) and multimers. In addition, a smaller species (~10 kDa) was observed in the highly concentrated fractions (lanes 8–9) and could correspond to a truncated or a degraded fragment of the fusion protein containing the N-terminal fusion tag.

Automated and gravity-driven drop columns were both used and the automated method was preferred due to scale of the purification. As expected an excess of protein to the column resulted in preparations of higher purity, as non-specific binding occurs when the resin binding sites are not fully occupied. Introducing a washing step using Buffer C, greatly reduced the amount of impurities. Although impurities appear faint on SDS PAGE gels, their absorption at 280 nm was sufficient to be seen on a chromatograph. After this first step of purification, the yield of purified denatured DI fusion protein was around 125–150 mg of protein/ l of medium when quantified in guanidine.

### In vitro folding of DI fusion protein and IMAC purification in native conditions

Two methods were compared for the in vitro folding of DI fusion protein. The first method was stepwise addition of ~1.0 mg (at a concentration of >10 mg/ml) of denatured protein into 100 ml of Buffer E while stirring at 4 °C; a total of 4 or 5 20-μl additions were made. The second method was continuous automated addition of protein using a syringe pump injecting the DI fusion protein (at a concentration >10 mg/mL) in the refolding buffer with a speed of 300 μL/h. The automated continuous addition method resulted in less protein aggregation after overnight incubation. The maximum yield of soluble protein obtained by continuous addition was 150 mg in 100 ml of Buffer E. Denatured protein concentration during folding was critical for the final yield of soluble protein, protein folded at concentrations lower than 10 mg/mL aggregated overnight.

The soluble DI fusion protein was then concentrated and dialysed against Buffer F prior to IMAC-purification in non-denaturing conditions. Avoiding high protein concentrations at this stage was critical and a concentration for folded fusion protein of 2 to 2.5 fold was found to be optimal. IMAC purification at this stage was very successful for the removal of impurities (Fig. 3, 10 kDa band), misfolded species and aggregates. Yield after IMAC purification was between 50 and 75 mg per litre of expression medium.

### Fusion tag cleavage, purification of untagged DI and endotoxin removal

**Fig. 4:**
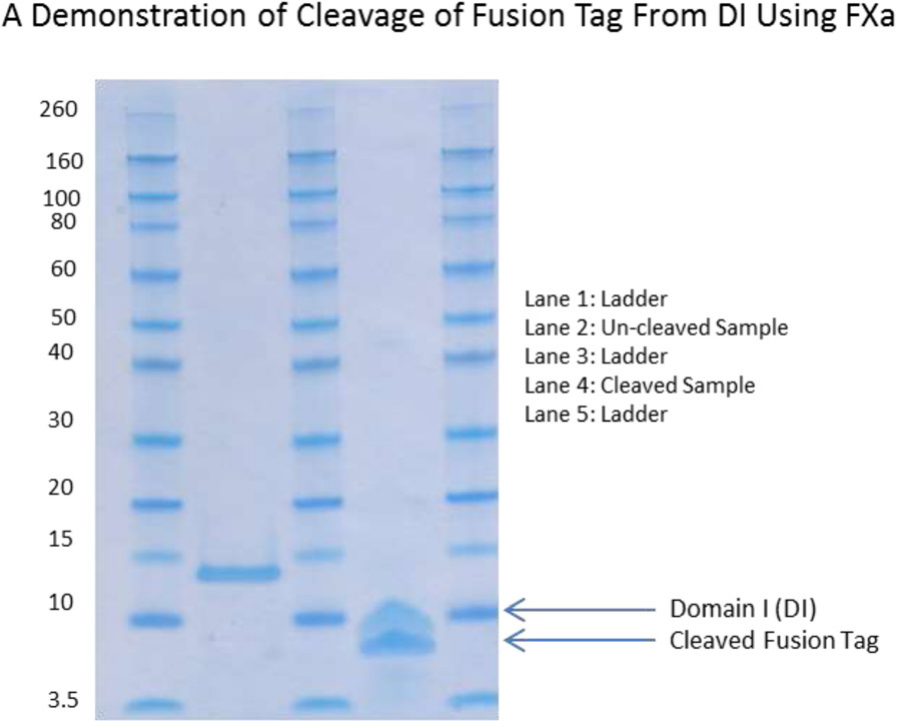
An SDS PAGE gel depicting purified DI fusion protein before FXa cleavage (Lane 2) and native DI and released fusion tag after FXa cleavage (labelled accordingly)

**Fig. 5:**
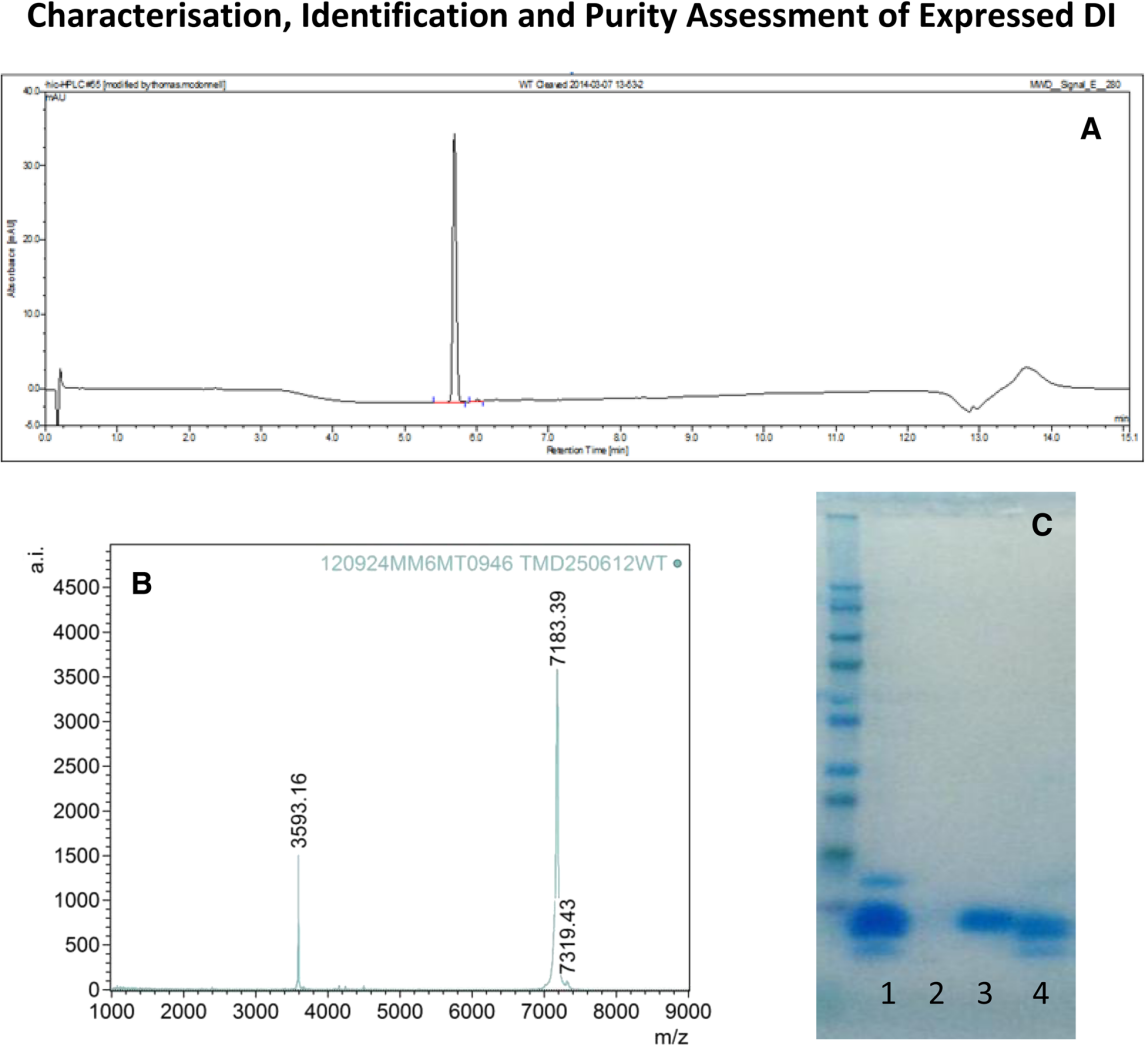
Characterisation of various DI batches by (**a**) RP-HPLC (C8) analysis, (**b**) MALDI-TOF and (**c**) SDS-PAGE Lane (1) Fusion protein, Lane (3) purified DI and Lane (4) cleaved fusion Tag. Lane 2 is blank

Removal of the N-terminal tag was performed by addition of restriction protease FXa to the fusion protein at a ratio of 1:200 and incubation overnight at 22 °C. The digested sample was then analysed by SDS-PAGE to confirm the completion of the digestion (Fig. 4). After overnight digestion the cleavage of the fusion protein was complete and SDS-PAGE analysis showed 2 bands (Fig. 4, lane 2), corresponding to the native DI (~8 kDa) and the fusion tag (~6 kDa). A final step of purification by cation exchange was performed and yielded >99 % pure monomeric DI as judged by SDS-PAGE (Fig. 5c) and reverse phase chromatography (Fig. 5a).

The concentration of protein for endotoxin removal was 0.3–0.6 mg/mL and required the addition of 1 mM extra CaCl_2_ for optimal yield. Endotoxin removal was successful down to a level of < 1 EU/100 μg.

### DI protein characterisation by mass spectroscopy and an activity assay

DI was characterised by molecular weight determination using MALDI-TOF mass spectroscopy. The mass spectrum of the full-length protein displayed an m/z value of 7183.39 which is in agreement with the expected m/z for the [M + H] + species of 7187.59, the mass error observed is within the calibration limitation given the small molecular weight of the protein.

**Fig. 6:**
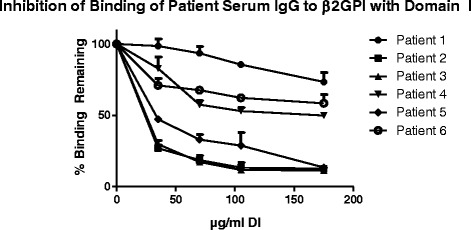
Competitive Inhibition assay. Sera from six patients were tested using the purified Domain I at various concentrations. Values plotted are an average of 2 experiments, SEM plotted above each point

For the competitive inhibition ELISA, Serum samples were obtained from six patients with APS. Three were male, all were Caucasian and the mean (SD 13.9) age was 47. All six patients had suffered vascular thrombosis and two patients had suffered foetal losses (1×2, 1×3). Importantly, serum from all six patients had been found to have very high binding to β2GPI in a solid phase ELISA assay. Figure 6 shows that of six serum samples 4 were inhibited by 50 % or more and two by 90 % (at 175 μg/ml patient 1–6: 73.3, 12.5, 11.3, 49.8, 13.4, 58.3 %).

## Conclusions

Although other methodologies such as mammalian or yeast expression may offer other benefits, such as post-translational modification or secretion of soluble product post-expression into medium, *E.Coli* offers many advantages to the lab scale scientist. It allows simple optimisation, is flexible, robust and can be translated to a GMP environment.

In this paper we present a protocol for the preparation of biologically active endotoxin-free DI expressed as inclusion bodies in *E. coli*. Denatured DI was purified by IMAC in guanidine hydrochloride, reduced with TCEP, and folded in vitro by dilution into a buffer containing 0.6 M arginine as aggregation inhibitor and cysteine/cysteine to promote the rearrangement of disulphide bonds. After further purification and fusion tag removal, the maximal yield was 50–75 mg per L of expression media. This represents a 20-fold increase on previous *E. coli* expression protocols for DI using in vivo folding by periplasmic localisation (750 μg/L [36]) or in vitro folding (~4 mg/L [37]).

The most vital stages of production, resulting in an increased yield, were the bacterial pellet collection, folding by continual injection and general automation. Replacing LB with TB resulted in an increase in yield but primarily due to an increased cell density rather than expression itself. This increased cell density led to a thicker lysate with more DNA, however, addition of DNAses to the lysate and extensive washing of the bacterial pellet reduced the viscosity and allowed a better purification process. These washes also reduced endotoxin levels during the purification process and resulted in a higher purity product. The instigation of automated refolding via the syringe pump increased yields by almost double and, when this was combined with a slower and less harsh dialysis protocol, optimal yields were obtained. It is generally accepted that aggregation occurs only between partially folded species and that the presence of folded protein does not result in increased aggregation [38], therefore gradual and very slow addition of protein to folding buffer reduces the effective concentration of unfolded species at any given moment. Similarly, the same can be said of the dialysis method. Automation of protein dilution also increased yields minimising the number of handling steps and human error. Interestingly, minimisation of the total protein concentration during the dialysis steps after folding also contributed to increase final protein yield, perhaps because a fraction of the protein is still partially unfolded at this stage.

Suggested future alterations to the method would include the introduction of an auto inducing culture [39]. Auto induction is a complex process but with the correct optimisation it may be used to increase the automation of the method, however, insufficient optimisation risks a decrease in yield. Despite the positive benefits of the new system we have described- increased expression, efficient folding and high purity- the method does have potential limitations. Preliminary attempts to use on-column refolding failed as the protein fell out of solution and stripped the column suggesting that alternative methods of folding may not be suitable during scale-up. On the other hand, only two methods of on-column refolding were tried and both utilised a 5 mL HisTrap IMAC column and further investigation with other chemistries may improve the technique. The major limiting factor of the method is the large volume of buffer required for folding. Similarly, the manual process of grinding the cells is a drawback which could easily be improved with equipment such as a French press. It is therefore possible that further optimisation may improve protein yield even more.

Further improvements to this methodology are necessary for scaling up and automation. The major limitation at this stage is the need to concentrate protein solutions in several points during the process. The introduction of an on-column protein folding method would ameliorate this drawback. Unfortunately, our preliminary attempts to implement such a methodology in the case of DI have failed; presumably because of the very poor intrinsic protein solubility of unfolded DI, which resulted in rapid protein aggregation as soon as the concentration of guanidine was reduced. An alternative and more promising approach would be to substitute protein concentration by centrifugal ultracentrifugation by ion exchange chromatography or tangential flow filtration steps. Another change in the process required for scaling up is the replacement of cell lysis by sonication for high pressure disruption or by chemical methods. It is also possible that yields can also be increased by optimising and/or altering the composition of the folding buffer, as a great variety of potential aggregation inhibitors has been described in the literature [38].

Regarding the activity of the protein, the inter patient variability in susceptibility to inhibition by DI is likely due to the fact that some patients possess anti-β2GPI antibodies that interact with other domains (DII-V) and is entirely consistent with results from previous experiments using DI expressed in insect cells [29].

This methodology demonstrates a relatively simple, inexpensive, reproducible and semi-automated bench top method for the expression, folding and purification of human DI up to a 0.1–0.15 g scale. This system opens the possibility for the development of DI as a potential therapeutic agent for APS.
